# New Hepatitis E Virus Genotype in Camels, the Middle East

**DOI:** 10.3201/eid2006.140140

**Published:** 2014-06

**Authors:** Patrick C.Y. Woo, Susanna K.P. Lau, Jade L.L. Teng, Alan K. L. Tsang, Marina Joseph, Emily Y.M. Wong, Ying Tang, Saritha Sivakumar, Jun Xie, Ru Bai, Renate Wernery, Ulrich Wernery, Kwok-Yung Yuen

**Affiliations:** State Key Laboratory of Emerging Infectious Diseases, Hong Kong, China (P.C.Y. Woo, S.K.P. Lau, K.-Y. Yuen);; The University of Hong Kong, Hong Kong (P.C.Y. Woo, S.K.P. Lau, J.L.L. Teng, A.K.L. Tsang, E.Y.M. Wong, Y. Tang, J. Xie, R. Bai, K.-Y. Yuen);; Central Veterinary Research Laboratory, Dubai, United Arab Emirates (M. Joseph, S. Sivakumar, R. Wernery, U. Wernery)

## Abstract

In a molecular epidemiology study of hepatitis E virus (HEV) in dromedaries in Dubai, United Arab Emirates, HEV was detected in fecal samples from 3 camels. Complete genome sequencing of 2 strains showed >20% overall nucleotide difference to known HEVs. Comparative genomic and phylogenetic analyses revealed a previously unrecognized HEV genotype.

Hepatitis E virus (HEV) belongs to the family *Hepeviridae* and genus *Hepevirus*. Among humans worldwide, HEV is the most common cause of acute viral hepatitis. The disease is generally self-limiting, but mortality rates are high among pregnant women and young infants. Chronic HEV infection is a problem for immunocompromised patients, such as those who have received a solid organ transplant and those with HIV infection. In addition to humans, HEV has been found in the other mammals: pigs, boar, deer, rodents, ferrets, rabbits, mongoose, bats, cattle, sheep, foxes, minks, and horses (*1**–**3*). Among the 4 known HEV genotypes, HEV1 and HEV2 infect only humans; whereas, HEV3 and HEV4 can infect humans, pigs, and other mammals. Human infections with HEV3 and HEV4 have been associated with consumption of raw or undercooked pork or game meat (*4*). Traditionally, HEV infection is mainly transmitted through water contaminated with infected feces. Since water supplies and sanitary infrastructures have been improved, animals have become a major source of human HEV infection. We detected HEV in fecal samples from dromedary camels in the Middle East.

## The Study

As part of a molecular epidemiology study, 203 fecal samples from 203 adult dromedaries (*Camelus dromedarius*) were submitted to the Central Veterinary Research Laboratory in Dubai, United Arab Emirates, over a 7-month period (January–July 2013). RNA extraction and reverse transcription were performed, as described, to detect other positive-sense single-stranded RNA viruses (*5**,**6*). Screening for HEV was performed by PCR amplification of a 284-bp fragment of open reading frame (ORF) 2 in HEV; specific primers used were 5′-TTTATTCTCGTCCAGTCGTTTC-3′ and 5′-GTCAGTGGAGGACCCATATGT-3′, designed from sequence information from our metagenomic study (P.C.Y. Woo et al., unpub. data). PCR was performed according to previously described conditions (*7*); annealing temperature were set at 50°C. DNA sequencing and quantitative real-time reverse transcription PCR were also performed as described (*8*). Using strategies we have reported for other positive-sense single-stranded RNA viruses, we performed complete-genome sequencing on 2 HEV-positive samples (*5**,**6*). Comparative genomic analysis was performed as described (*9*). Phylogenetic analysis was conducted in MrBayes5D version 3.1.2 (www.fifthdimension.jp/products/mrbayes5d/) by using an optimal substitution model with 1 million Markov chain Monte Carlo generations; sampling was conducted every 100 generations with a burn-in of 25,000. The substitution model was selected on the basis of the corrected Akaike information criterion by ProtTest version 2.4 (http://darwin.uvigo.es/software/prottest.html).

Reverse transcription PCR for a 284-bp fragment in ORF2 of this HEV, which we named dromedary camel HEV (DcHEV), was positive for 3 fecal samples; viral loads were 3.7 × 10^5^, 4.5 × 10^5^, and 3.2 × 10^7^ copies/mL. Complete-genome sequence data for 2 DcHEV strains (GenBank accession nos. KJ496143–KJ496144) revealed that the genome size was 7,220 bases and had a G+C content of 55% (Table). Overall, the DcHEV genomes differed from all other HEVs by >20% nt (Technical Appendix Table).

**Table:** Comparison of genomic organization of HEV genotypes and isolates*

HEV (GenBank accession no.)	Genome length, nt	GC content, %	5′ UTR, nt	ORF1, aa	ORF2, aa	ORF3, aa	3′ UTR, nt
DcHEV-178C (KJ496143)†	7,220	55.1	39	1,698	660	113	66
DcHEV-180C (KJ496144)‡	7,219	54.4	39	1,698	660	113	66
HEV1 (M73218)	7,194	58.1	27	1,693	660	114	65
HEV2 (M74506)‡§	>7,170	56.5	NA	1,691	659	114	74
HEV3 (AB089824)	7,244	55.3	25	1,709	660	113	72
HEV4 (AJ272108)	7,232	54.4	25	1,707	658	112	68
Rabbit HEV (FJ906895)‡	7,283	55.5	26	1,722	660	113	71
Germany rat HEV (GU345042)	6,948	57.8	10	1,636	644	102	65
Vietnam rat HEV (JX120573)	6,927	56.6	10	1,629	644	102	65
Ferret HEV (JN998606)	6,841	53.8	12	1,596	654	108	65
Wild boar HEV novel unclassified genotype (AB602441)	7,246	57.0	25	1,709	660	112	70
Bat HEV (JQ001749)	6,767	51.8	33	1,580	637	137	77
Avian HEV genotype 1 (AM943647)§	>6,627	55.1	NA	>1,531	606	87	123
Avian HEV genotype 2 (AY535004)	6,654	55.5	24	1,531	606	87	127
Avian HEV genotype 3 (AM943646§	>6,631	55.6	NA	1,532	606	87	126
Avian HEV novel unclassified genotype (JN997392)§	>6,543	55.7	NA	>1,515	606	87	NA
Cutthroat trout HEV (HQ731075)	7,269	49.7	100	1,707	634	225	76

*HEV, hepatitis E virus; UTR, untranslated region; ORF, open reading frame; DcHEV HEV from dromedary camel; NA, not available because of incomplete genome. †Assuming the third AUG of ORF2 is the start codon. ‡Assuming the third AUG of ORF3 is the start codon. §Near-complete genome.

**Figure 1:**
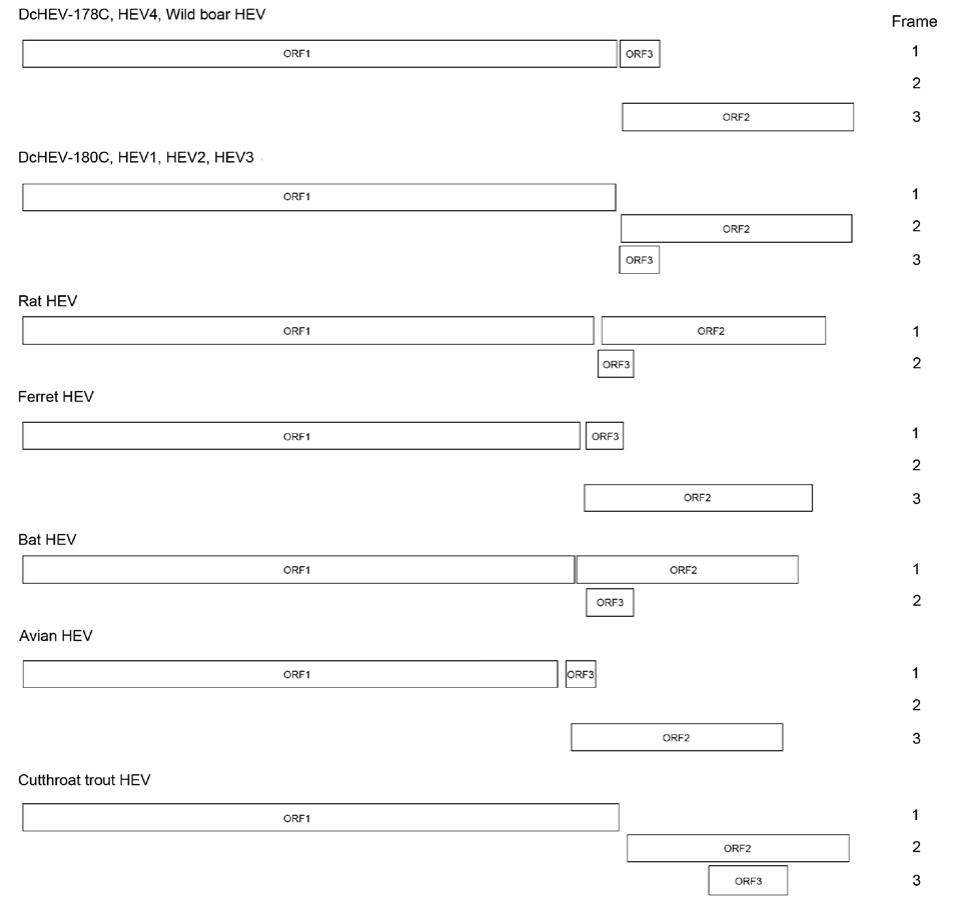
Predicted genomic organization of hepatitis E virus (HEV) from dromedary camel (DcHEV) and other HEVs, considering the reading frame of open reading frame (ORF) 1 as frame 1.

The DcHEV genome contained 3 major ORFs (Table, Figure 1). ORF1 polyprotein contained motifs consistent with a methyltransferase, a peptide containing a Y domain, a papain-like cysteine protease, a peptide with a hypervariable region (HVR), a helicase, and an RNA-dependent RNA polymerase. Also present in DcHEV were conserved sequences TLYTRTWS and RRLLXTYPDG, which bound the HVR of HEV1–4 and of 2 recently discovered wild boar HEV strains (*10**, **11*) but not the HVR of ferret, rat, bat, avian or cutthroat trout HEVs. A conserved motif, (T/V)SGFSS(D/C)F(S/A)P, immediately preceding the HVR of only HEV3 and HEV4 was present in DcHEV as VSGFSSDFAP. The relative excess of proline and serine observed in the HVR of all other HEVs was also observed for DcHEV. For DcHEV strain 178C, ORF2 began at nt 5172, similar to what is found for HEV4 and wild boar HEV, with an insertion of a single nucleotide (U) at nt 5146, and ended at nt 7154, encoding a capsid protein of 660 aa (Technical Appendix Figure) (*11**–**13*). As for DcHEV strain 180C, because of the lack of the U insertion as in HEV1, HEV2, and HEV3, ORF2 began at nt 5171 (Technical Appendix Figure). For DcHEV strain 178C, similar to HEV4 and the 2 recently discovered wild boar HEV strains (*11*), ORF3 began at nt 5161 (Technical Appendix Figure) and ended at nt 5502, encoding a small phosphoprotein of 113 aa with a multifunctional C-terminal region. As for DcHEV strain 180C, because of the lack of the U insertion as in HEV1, HEV2, and HEV3, ORF3 began at nt 5160. The conserved *cis*-reactive element (UGAAUAACAUGU) located upstream of ORF2 and ORF3 in both strains might serve as promoter for the synthesis of the subgenomic mRNA for these 2 ORFs.

**Figure 2:**
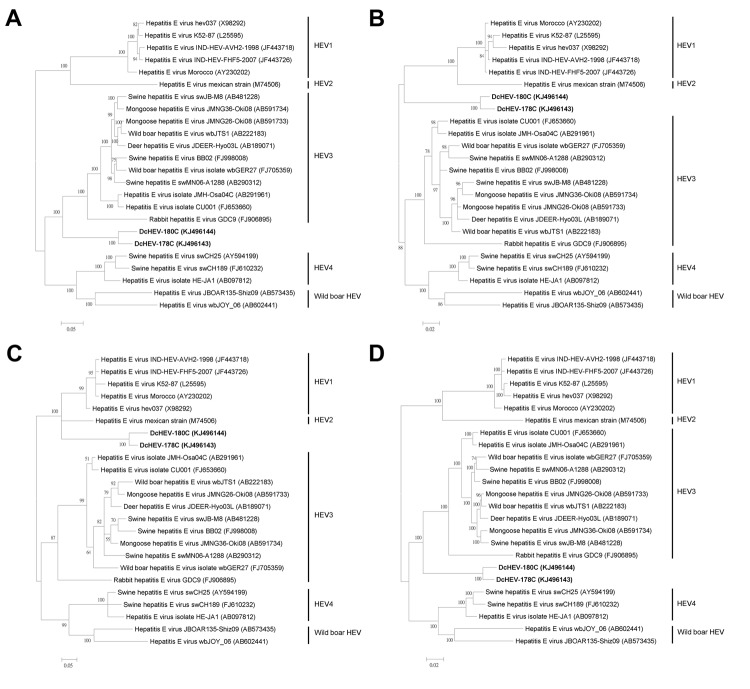
Phylogenetic analyses of open reading frame (ORF) 1 (A), ORF2 (B), ORF3 (C), and ORF1/ORF2 proteins, excluding the hypervariable region (HVR) (D) of hepatitis E virus (HEV) from dromedary camels (DcHEV). The trees were constructed by using Bayesian methods of phylogenetic reconstruction (www.fifthdimension.jp/products/mrbayes5d/), and ProtTest-suggested JTT+I+G+F, MtMam+I+G+F, HIVw+I+G+F, and JTT+I+G+F (http://darwin.uvigo.es/software/prottest.html) are the optimal substitution models for ORF1, ORF2, ORF3, and concatenated ORF1/ORF2 excluding HVR, respectively. For this analysis we included amino acid positions 1698, 660, 113, and 2282 in ORF1, ORF2, ORF3 and concatenated ORF1/ORF2 excluding HVR, respectively. For ORF2 and concatenated ORF1/ORF2 excluding HVR, the scale bars indicate the estimated number of substitutions per 50 aa. For ORF1 and ORF3, the scale bars indicate the estimated number of substitutions per 20 aa. Boldface indicates the 2 strains of DcHEV with complete genomes sequenced in this study.

Phylogenetic trees constructed by using ORF1, ORF2, ORF3, and concatenated ORF1/ORF2 excluding the HVR showed that DcHEV was clustered with different HEVs in different phylogenetic trees (Figure 2). For ORF1 and concatenated ORF1/ORF2 excluding the HVR, DcHEV was clustered with HEV3; but for ORF2 and ORF3, DcHEV was clustered with HEV1 and HEV2. Recombination analysis performed by using bootscan revealed no obvious and definite site of recombination, similar to what we observed in previous studies for other viruses (*14*), although different regions of the DcHEV genome might be more similar to different genotypes of HEV (data not shown).

## Conclusions

We discovered HEV in dromedaries from the Middle East and named the virus DcHEV. In a recent study conducted in Dubai, HEV accounted for 40% of cases of acute hepatitis in humans (*15*). Although HEV is a major pathogen in the Middle East, sequence data for HEVs in the Arabian Peninsula are limited. The study reported here revealed that 1.5% of the adult dromedary fecal samples showed evidence of DcHEV RNA. Because humans come in close contact with dromedaries, our finding of DcHEV in dromedaries indicates a previously unknown potential reservoir and source of HEV infection for humans.

Comparative genomic and phylogenetic analyses showed that DcHEV probably represents a previously unrecognized HEV genotype. The conserved motif preceding the HVR in ORF1 resembled those found in HEV3, HEV4, and the 2 recently discovered wild boar HEV strains. Although phylogenetically ORF1 of DcHEV was clustered with HEV3, ORF2 and ORF3 of DcHEV were clustered with HEV1 and HEV2. Of note, ORF2 and ORF3 of the 2 DcHEV strains with complete genomes sequenced in this study resembled those of different HEV genotypes. The presence of a U insertion downstream to the second possible start codon for ORF2 (AUG2) in DcHEV strain 178C resembled the presence of a U insertion in HEV4 and wild boar HEV, leading to 3 possible start codons for its ORF2 but 1 possible start codon for its ORF3; whereas, the lack of this U insertion downstream to AUG2 in DcHEV strain 180C resembled the lack of U insertions in HEV1, HEV2 and HEV3, leading to only 1 possible start codon for its ORF2 but 3 possible start codons for its ORF3. To our knowledge, this presence or absence of such a U insertion in different strains of the same HEV has never been observed in other HEV genotypes and is unique to DcHEV. Although different regions of the DcHEV genome possessed characteristics associated with different kinds of HEV, no significant recombination was detected between DcHEV and the other HEVs. Because different regions of the genomes of DcHEV resembled those of different HEV genotypes, and even the genomes of different strains of DcHEV resembled those of different HEV genotypes, we propose that DcHEV should constitute a new HEV genotype.

Technical Appendix Comparison of nucleotide and deduced amino acid sequence identities of hepatitis E virus (HEV) from dromedary camels (DcHEV) and other genotypes of HEV; alignment of nucleotide sequences showing potential start codons for open reading frames 2 and 3 in DcHEV and other HEVs.
