# Supplementary material for: New Hepatitis E Virus Genotype in Camels, the Middle East
Source: Emerg Infect Dis. 2014 Jun;20(6):1044–8. doi: 10.3201/eid2006.140140 (PMC4036782; doi:10.3201/eid2006.140140)
Supplement: Technical Appendix — Comparison of nucleotide and deduced amino acid sequence identities of hepatitis E virus (HEV) from dromedary camels (DcHEV) and other genotypes of HEV; alignment of nucleotide sequences showing potential start codons for open reading frames 2 and 3 in DcHEV and other HEVs. [file 14-0140-Techapp-s1.pdf]

DOI: http://dx.doi.org/10.3201/eid2006.140140

New Hepatitis E Virus Genotype in Camels, the Middle East

Technical Appendix

Technical Appendix Table. Comparison of nucleotide and deduced amino acid sequence identities of DcHEV and other genotypes of HEV

HEV genotypes/strains (GenBank accession no.)	DcHEV-178C (KJ496143)						
	Nucleotide identity (%)				Amino acid identity (%)		
	Entire genome	ORF1	ORF2	ORF3	ORF1	ORF2	ORF3
DcHEV-180C (KJ496144)	86.1	85.0	88.5	94.7	94.9	98.0	94.7
HEV1 (L08816)	74.2	72.6	79.3	86.1	82.1	89.4	83.3
HEV2 (M74506)*	73.7	72.4	78.5	85.2	81.8	88.6	79.8
HEV3							
Human HEV (FJ653660)	75.4	74.3	79.3	85.4	87.2	91.1	78.8
Mongoose HEV (AB591734)	75.8	74.4	79.9	86.0	86.5	90.9	79.6
Deer HEV (AB189071)	76.1	75.4	78.9	85.4	86.9	90.8	77.9
Rabbit HEV (FJ906895)	74.1	72.3	78.6	83.3	83.2	88.6	76.1
HEV4 (AJ272108)	74.6	73.1	78.9	81.4	84.7	88.5	71.9
Wild boar HEV novel unclassified genotype							
JBOAR135-Shiz09 (AB573435)	74.8	73.1	79.4	81.9	83.6	90.6	70.2
wbJOY_06 (AB602441)	74.0	72.9	77.2	80.7	82.5	88.8	68.4
Rat/Ferret HEV							
Germany rat HEV (GU345042)	56.5	55.3	60.2	53.9	50.1	55.5	27.8
Vietnam rat HEV (JX120573)	56.4	55.3	60.1	53.9	50.8	56.1	32.2
Ferret HEV (JN998606)	56.3	54.9	60.0	52.4	50.8	56.7	30.3
Bat HEV (JQ001749)	54.1	53.4	56.2	45.6	43.7	47.2	20.9
Avian HEV							
Avian HEV genotype 1 (AM943647)*†	52.6	52.2	53.4	43.1	43.2	45.3	25.0
Avian HEV genotype 2 (AY535004)	52.8	52.6	52.7	43.7	43.7	45.5	25.0
Avian HEV genotype 3 (AM943646)*	52.6	52.2	53.2	44.9	42.9	45.4	23.1
Avian HEV novel unclassified genotype (JN997392)*†	52.1	51.7	53.0	45.6	43.3	45.4	26.4
Cutthroat trout HEV (HQ731075)	48.3	49.0	46.8	34.6	27.4	20.3	14.6

*Near-complete genome.
†Partial ORF1.

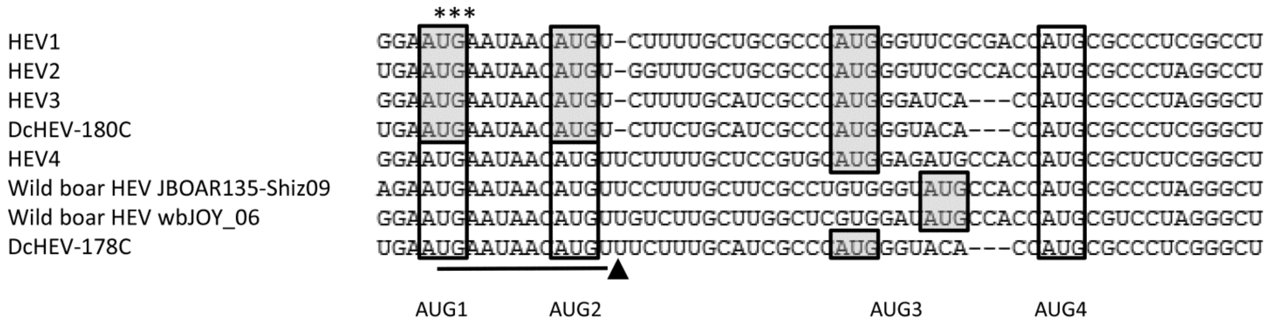

Technical Appendix Figure. Alignment of nucleotide sequences showing potential start codons for ORF2 and ORF3 in DcHEV and other HEVs. Potential start codons of ORF3 are indicated by shaded boxes, and those of ORF2 by open boxes. The inserted U residue is marked with a closed triangle. The stop codon of ORF1 is indicated by asterisks. The conserved *cis*-reactive element with the sequence UGAAUAACAUGU present in HEV1–4 and wild boar HEV is underlined.
